# Hearing Loss, Tinnitus, Hyperacusis, and Diplacusis in Professional Musicians: A Systematic Review

**DOI:** 10.3390/ijerph15102120

**Published:** 2018-09-26

**Authors:** Arianna Di Stadio, Laura Dipietro, Giampietro Ricci, Antonio Della Volpe, Antonio Minni, Antonio Greco, Marco de Vincentiis, Massimo Ralli

**Affiliations:** 1 Otolaryngology Department, University of Perugia, 06123 Perugia, Italy; ariannadistadio@hotmail.com (A.D.S.); giampietro.ricci@unipg.it (G.R.); 2 Highland Instruments, Cambridge, MA 02238, USA; lauradp@mit.edu; 3 Santobono-Pousillipon Hospital, Cochlear Implant Center, 80129 Naples, Italy; antoniodellavolpe@yahoo.it; 4 Department of Sense Organs, Sapienza University of Rome, 00185 Rome, Italy; antonio.minni@uniroma1.it (A.M.); antonio.greco@uniroma1.it (A.G.); 5 Department of Oral and Maxillo-Facial Science, Sapienza University of Rome, 00185 Rome, Italy; marco.devincentiis@uniroma1.it; 6 Center for Hearing and Deafness, University at Buffalo, Buffalo, NY 14260, USA

**Keywords:** hearing loss, noise induced hearing loss, musicians, pop-rock, classic, tinnitus, hyperacusis, diplacusis

## Abstract

Professional musicians (PMs) are at high risk of developing hearing loss (HL) and other audiological symptoms such as tinnitus, hyperacusis, and diplacusis. The aim of this systematic review is to (A) assess the risk of developing HL and audiological symptoms in PMs and (B) evaluate if different music genres (Pop/Rock Music—PR; Classical Music—CL) expose PMs to different levels of risk of developing such conditions. Forty-one articles including 4618 PMs were included in the study. HL was found in 38.6% PMs; prevalence was significantly higher among PR (63.5%) than CL (32.8%) PMs; HL mainly affected the high frequencies in the 3000-6000 Hz range and was symmetric in 68% PR PMs and in 44.5% CL PMs. Tinnitus was the most common audiological symptom, followed by hyperacusis and diplacusis. Tinnitus was almost equally distributed between PR and CL PMs; diplacusis was more common in CL than in PR PMs, while prevalence of hyperacusis was higher among PR PMs. Our review showed that PR musicians have a higher risk of developing HL compared to CL PMs; exposure to sounds of high frequency and intensity and absence of ear protection may justify these results. Difference in HL symmetry could be explained by the type of instruments used and consequent single-sided exposure.

## 1. Introduction

Hearing loss (HL) can follow exposure to loud sounds; noise-induced HL is the second most common cause of HL, and accounts for about 16% of disabling HL in the adult population worldwide [1,2]. Chronic exposure to noise causes a progressive destruction of inner and outer hair cells in the cochlea following oxidative stress, metabolic exhaustion, and ischemia [3,4,5]. Noise-induced HL can follow work-related and recreational noise exposure [6,7,8,9,10] with significant impact on quality of life [11,12].

Tinnitus, defined as the perception of sound without an external auditory stimulus, is a condition affecting 10–25% of the adult population, with moderate-to-severe consequences on daily activities and quality of life [13,14]. Risk factors for tinnitus include hearing loss, exposure to loud sounds, and increasing age [15,16]; tinnitus ranges from 35 to 77% in subjects with noise-induced HL [17,18]. Furthermore, subjects often report worsening of tinnitus with stress; therefore, workers subject to high work-related stress may have an increased risk of tinnitus [19,20].

Hyperacusis is defined as a reduced tolerance to sounds of average intensity, sometimes accompanied by painful sensitivity to ordinary environmental sounds, with perceptual, psychological, and social dimensions [21]. Hyperacusis is often associated tinnitus [22,23].

Diplacusis is a term used to describe an anomaly whereby the same tone is perceived as having a different pitch depending on whether it is presented in the right or in the left ear of the same listener [24,25]; evidence suggests a higher prevalence of diplacusis in individuals with hearing loss, especially asymmetric [24,25].

Music, at the both entertainment and professional level, can induce HL and other audiological symptoms such as tinnitus, hyperacusis, and diplacusis [9,26]; professional musicians (PMs) are exposed to high intensity sounds for a prolonged time during the day [27]. Many studies have focused on the prevalence of HL among PMs and the degree of hearing loss among them [28,29,30]; however, the extent to which the musical genre affects the risk of developing hearing loss in PM remains unclear [30,31]. Different music genres such as Pop/Rock (PR) or classical (CL) music entail different levels of noise exposure, which in turn depend on several factors including the type and number of instruments that are played at the same time and the intensity of the sound that is generated [27,28,29,30,31].

The aims of this systematic review of the literature are to (A) assess the risk of developing HL and audiological symptoms (tinnitus, hyperacusis, diplacusis) in PMs and (B) evaluate if different music genres expose PMs to a different level of risk of developing these conditions.

## 2. Materials and Methods

### 2.1. Search Strategy

This systematic review followed the Preferred Reporting Items for Systematic Review and Meta-Analyses (PRISMA) guidelines [32]. Two researchers independently searched PubMed, Scopus, and Google Scholar (until 06/30/2018) using the following keywords: “hearing loss”, “tinnitus”, “hyperacusis”, “music”, “classical music”, “rock music”, “pop music”, “musicians”, “orchestra”, “music student”, “professional musician”, “hearing threshold”, “music exposure”, and “temporary hearing loss”. Both researchers independently selected and reviewed the abstracts that included a minimum of two keywords with “hearing loss” and “music” as the principal ones. The selected articles were then thoroughly read.

### 2.2. Study Selection

All publication types from 1978 to April 2018, in English, French, or Italian only, were considered for analysis, including epidemiological, case control, prospective and retrospective studies. Studies on non-PMs were excluded; a PM was defined as a person for whom playing music was the primary occupation. Articles from the same author were thoroughly checked to avoid duplicates.

### 2.3. Data Extraction

A standardised electronic data extraction form was completed with pertinent information from each article. From the articles that were selected, the following details were extracted: study design, number and age of subjects, type of music played, type of instrument played, rehearsal time, degree of hearing loss, affected frequencies, hearing loss symmetry, presence of tinnitus, hyperacusis, and diplacusis. Hearing was considered symmetrical if mean thresholds for each ear occurred within 15 dB of each other.

### 2.4. Data Analysis

Meta-analyses require studies of similar comparison reporting identical outcome measures. The studies included in the present review revealed a considerable heterogeneity in study design, type of exams performed, length of observation period, and type of outcome measures. Therefore, a well-defined meta-analysis was not applicable, and a systematic review of the literature was preferred.

All data were added to a database; data related to the PR and CL genres were compared. Prevalence of symptoms was calculated as a percentage. Nominal data between PR and CL PMs, namely presence or absence of hearing loss, presence or absence of tinnitus, presence or absence of diplacusis were compared using chi-square (χ). Odds ratios were calculated for CL and PR PMs to quantify their risk of developing HL. Statistical significance level was defined at *p* < 0.05. 

## 3. Results

### 3.1. Articles and Subjects Included in the Systematic Review

**Figure 1:**
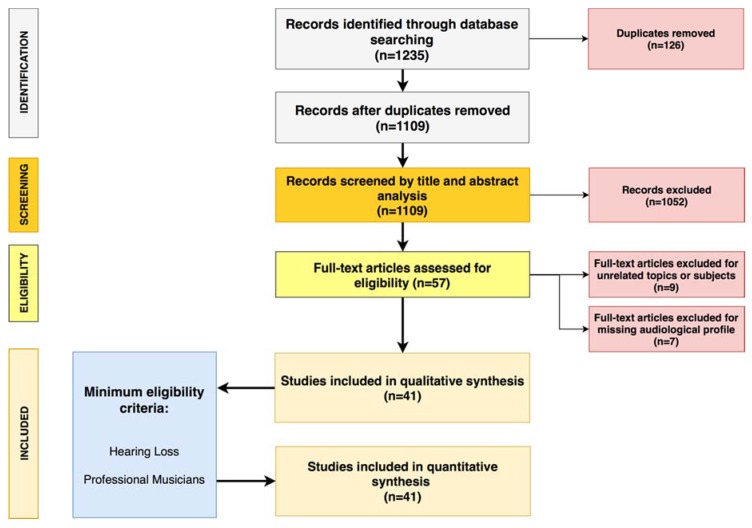
Preferred Reporting Items for Systematic Review and Meta-Analyses (PRISMA) diagram followed in the present review. The flow diagram depicts the flow of information through the different phases of the systematic review. It maps out the number of records identified, included and excluded, and the reasons for exclusions [32].

A total of 57 articles matching the inclusion criteria were identified. Among these, nine articles were excluded because they addressed other topics (*n* = 2) or involved different populations (non-professional musicians *n* = 3, singers *n* = 2, music technicians *n* = 1, general population *n* = 1), and seven articles were excluded because they were centered exclusively on risk assessment without including details on specific hearing disorders. Following this selection, 41 articles were included in the review (Figure 1).

Details of articles included in our review are summarized in Table 1.

Among these, 25 (60.9%) were prospective studies, 7 (17.1%) were case studies, 4 (9.8%) were cohort studies, 3 (7.4%) were longitudinal studies, 1 (2.4%) was a cross-sectional study, and 1 (2.4%) was a retrospective study. Twenty-six (63.5%) studies were conducted on CL PMs only, 11 (26.8%) on PR PMs only, and 4 (9.7%) on PR and CL PMs. Thirty-two (78.1%) studies reported details on the instruments played by the PM; in 9 studies (21.9%) the PMs were identified as PR or CL players but details about the instruments were not provided.

**Figure 2:**
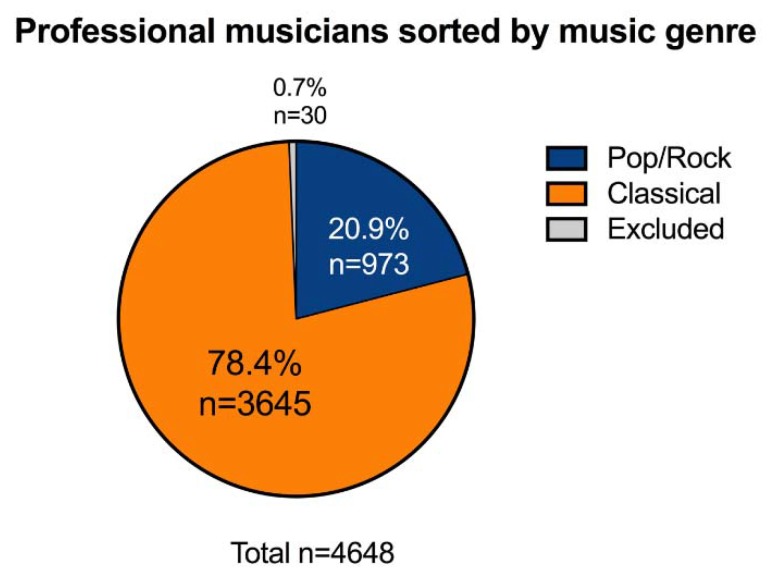
Distribution of professional musicians included in the review, divided by music genre (Pop/Rock; Classical).

The articles selected for the review included a total of 4648 PMs, 3645 CL PMs (78.4%) and 973 PR PMs (20.9%); 30 subjects (0.7%) were excluded due to lack of details on the music genre played (Figure 2).

Age for PR and CL PMs ranged from 16 to 69 years, with no significant differences between the groups (*p* = 0.78); 2914 (63.1%) of PMs included in the review were males; 1704 (36.9%) were females.

### 3.2. Hearing Loss and Audiological Symptoms

HL (Pure tone threshold >25 dB at any frequency in the 250–8000 Hz range) was investigated in 41/42 (97.6%) articles included in the review (4507 PMs) and was found in 1742 subjects (38.6%). PR PMs were more affected compared to CL PMs, with respectively 547/862 (63.5%) and 1195/3645 (32.8%) subjects; the odds ratio for PR was 1.3991 (CI 95%: 0.4044–0.5441). The difference was statistically significant (χ: *p* < 0.0001).

**Figure 3:**
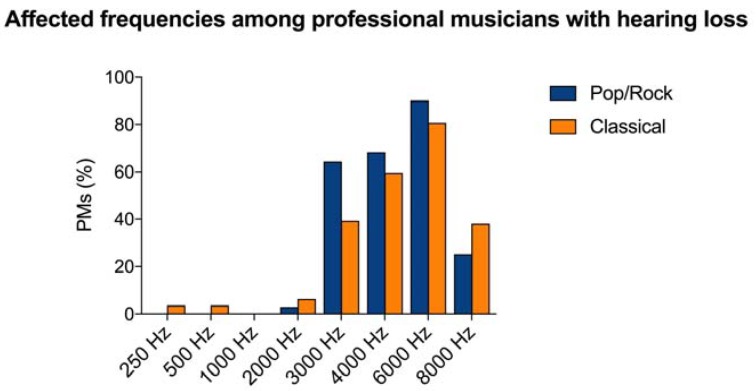
Affected frequencies among Pop/Rock and Classical professional musicians (PMs) with hearing loss included in the systematic review in the 250–8000 Hz frequency range. The most affected frequencies in both groups were 3000, 4000, and 6000 Hz.

HL affected prevalently the high frequencies in the range between 3000 and 6000 Hz. Among PR PMs, HL was reported for the 2000 Hz frequency in 16 (2.9%) subjects, 3000 Hz in 352 (64.3%), 4000 Hz in 370 (68.2%), 6000 Hz in 493 (90.1%), and 8000 Hz in 138 PMs (25.2%). In CL PMs, HL affected the 250 Hz frequency in 42 (3.5%) subjects, 500 Hz in 42 (3.5%), 2000 Hz in 74 (6.2%), 3000 Hz in 470 (39.3%), 4000 Hz in 712 (59.6%), 6000 Hz in 964 (80.7%), and 8000 Hz in 454 PMs (38%). Interestingly, no HL was reported for frequencies <2000 Hz for PR PMs. Details of HL in PMs are shown in Figure 3.

**Figure 4:**
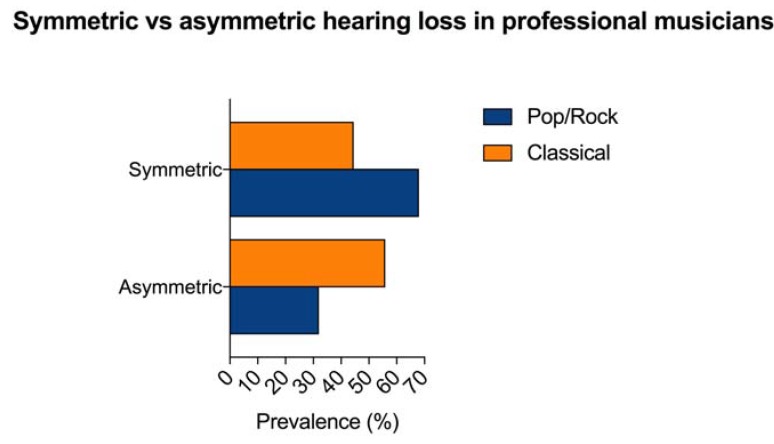
Percentage of symmetric and asymmetric hearing loss found in professional musicians (PMs) included in the systematic review. Symmetric HL was more common in Pop/Rock PMs, while asymmetric HL was more prevalent in Classical PMs. Data on symmetry were unavailable for 135 subjects.

HL was symmetric (thresholds for each ear within 15 dB of each other) in 835 PMs and asymmetric in 772 PMs. Characteristics on HL symmetry were not reported for 135 (7.7%) subjects (37 PR and 98 CL PMs). Symmetric HL was described in 347/510 (68%) PR PMs and in 488/1097 (44.5%) CL PMs; asymmetric hearing loss was present in 163 (32%) PR and in 609 (55.9%) CL PMs. CL PMs showed an odds ratio for asymmetric HL of 4.02 (CI 95%: 3.2574–4.9823). The difference between PR and CL PMs for asymmetric hearing loss was statistically significant (χ: *p* < 0.0001). Figure 4 shows the percentage of symmetric and asymmetric HL in PMs.

**Figure 5:**
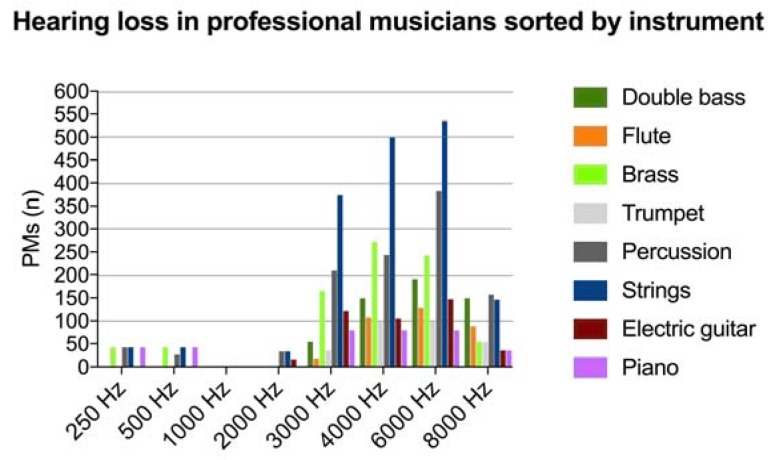
Number of professional musicians (PMs) with hearing loss in the 250–8000 Hz frequency range sorted by instrument played.

The most common instruments were double bass, brass, flute, trumpet, percussion, strings, electric guitar, and piano. Although some are more commonly used by PR or CL PMs, in many cases they were used simultaneously in both genres. The highest extent of hearing loss was reported in PMs using strings (*n* = 1628), followed by percussion (*n* = 1050), brass (*n* = 775), double bass (*n* = 543), electric guitar (*n* = 424), piano (*n* = 314), flute (*n* = 341), and trumpet (*n* = 284). Details of affected frequencies in PMs sorted by instrument played are shown in Figure 5.

Tinnitus was investigated in 17/41 (41.5%) studies including 2327 PMs (760 PR, 1567 CL) and was reported in 612 subjects (26.3%). Of those, tinnitus was found in 196/760 (25.8%) PR and in 416/1567 (26.5%) CL PMs. The odds ratio was 0.98 (CI 95%: 0.7616–1.131). No statistically significant difference was observed for tinnitus prevalence between CL and PR PMs (χ: *p* = 0.45).

Hyperacusis was evaluated in 8/41 studies (19.5%) including 791 PMs (288 PR, 503 CL) and was found in 172 musicians (21.7%). Of these, 77/288 (26.7%) were PR and 95/503 (18.9%) were CL PMs. The odds ratio was 1.56 (CI 95%: 1.1117–2.2095); the difference in the prevalence of hyperacusis between CL and PR was statistically significant (χ: *p*= 0.01) with increased risk for PR PMs.

Diplacusis was investigated in only 2 studies (4.5%) including 380 PMs and was found in 24 of them (6.3%), specifically in 4/139 (2.9%) PR and in 19/241 (7.9%) of CL PMs.

**Figure 6:**
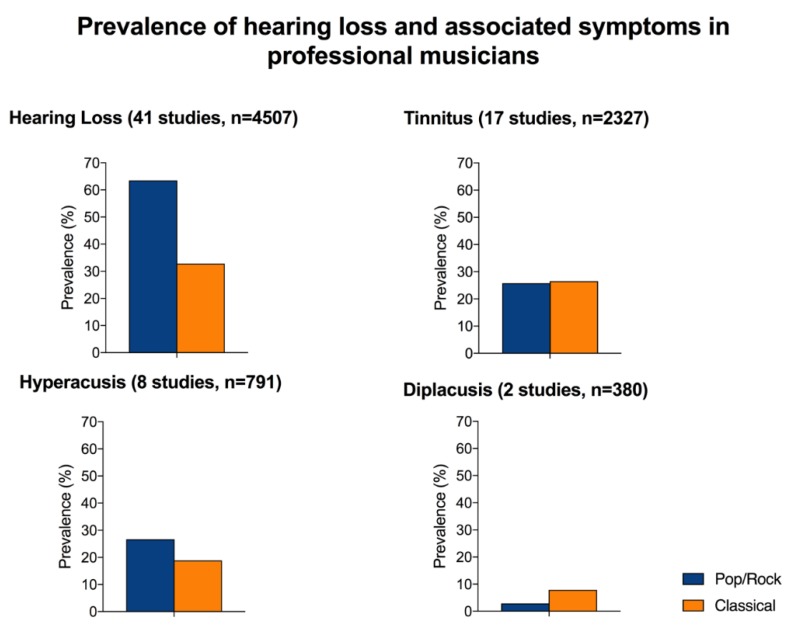
Prevalence of hearing loss tinnitus, hyperacusis, and diplacusis in Classical and Pop/Rock professional musicians included in the meta-analysis. For each condition, it has been indicated the number of studies that investigated it and the total number of patients included in these studies.

Tinnitus was equally prevalent in PR and CL PMs, while hyperacusis was more common in PR PMs and diplacusis in CL PMs. Figure 6 summarizes the prevalence of hearing loss, tinnitus, hyperacusis, and diplacusis in PR and CL PMs.

## 4. Discussion

Our systematic review of the literature showed that (A) 38.6% of PMs have some degree of HL; (B) PR PMs are at higher risk of developing HL than CL PMs; (C) the most affected frequencies in PMs are in the 3000–6000 Hz range; (D) CL PMs suffer from asymmetric HL significantly more than PR PMs; and (E) tinnitus was equally prevalent in PR and CL PMs, hyperacusis was more common in PR PMs and diplacusis in CL PMs.

### 4.1. Risk of Developing Hearing Loss Among PR and CL PMs

Our data shows that HL is common in PMs (38.6%), and PR PMs are at higher risk of developing HL compared to CL PMs. The presence of HL in PMs is a common finding and follows prolonged and high-intensity noise exposure, a condition that causes a progressive reactive oxygen and nitrogen species-mediated destruction of inner and outer hair cells in the cochlea following oxidative stress, metabolic exhaustion, and ischemia [3,4,5]. In the studies included in our review, HL affected mainly frequencies in the 3000–6000 Hz range, with no significant differences between PR and CL musicians.

The higher prevalence of HL in PR compared to CL PMs can be explained by different characteristics of the music genre, such as frequency and intensity of the instruments used, differences in habits of PMs and in environmental acoustic settings. Instruments produce sounds that span between different frequency ranges and emit sounds at different intensities. The frequencies generated by the instruments played is a key factor when assessing the risk of developing HL for PMs [27]; therefore, the musical genre may affect the characteristics of sounds PMs are exposed during practice [27]. Previous studies have shown that PR PMs are exposed to sounds of higher intensity compared to CL PMs, both during rehearsals and live performance [31], with an average exposure of 103 dB in PR versus 94 dB in CL PMs [27]; additionally, studies have described that PR PMs often play music without ear protection more commonly than CL PMs [28,29].

Furthermore, the introduction of new instruments such as the violin in PR music also plays a role, as the combination of high frequency-generating instruments and high intensity sound has been shown to increase risk of HL [27,70].

Future research should focus on evaluating the effects of sound in the central auditory pathways. In fact, although the role of peripheral damage in HL has been widely studied, recent research findings support the idea that noise can induce hearing loss also by acting directly on the central auditory pathways [71,72].

### 4.2. Characteristics of Hearing Loss Among PM

Our systematic review shows that CL PMs suffer from asymmetric HL significantly more than PR PMs. This difference can be explained by the instruments played by these professionals: in a classical music orchestra, a large portion of musicians play single-sided instruments, such as the violin (string) or the transverse flute; with these instruments the ear that is on the ipsilateral side (commonly the left for right handed musicians) is the most exposed to sound [65]. In PR PMs, the low incidence of asymmetric HL could be explained by prolonged exposure to high intensity sounds, such as those generated by electronic guitars, and the Larsen effect. Furthermore, the instruments commonly played by PR musicians (guitar, bass, drum) are symmetric and tend to equally expose both ears to sound.

### 4.3. Audiological Symptoms: Tinnitus, Hyperacusis, and Diplacusis

Our review shows a prevalence of tinnitus of 26.3% in PMs in the studies that investigated the symptom; it was almost equally distributed between CL and PR PMs. Tinnitus may follow audiological, somatic, or psychological conditions [13,73,74,75,76,77,78,79,80]; risk factors for tinnitus include HL and increasing age [15,16,81]. The elevate prevalence of HL found in PMs can explain the presence of tinnitus in these patients; however, there was no higher prevalence of tinnitus among PR PMs compared to CL PRs. This could be explained by the different number of studies that were considered to evaluate the prevalence of HL (*n* = 41) and tinnitus (*n* = 17); unfortunately, many studies did not evaluate the presence of tinnitus.

Hyperacusis [21] was the second most common symptom found in our review, and was more common in PR PMs compared to CL PMs. Hyperacusis may follow functional changes within the central nervous system and may be related to increased gain in the central auditory pathways and increased anxiety, mood disorders, or emotional response to sound [82,83]. The higher prevalence of hyperacusis in PR PMs could be related to changes in mood that can be elicited by the different music genres and the different stress levels. In fact, the volume of music can influence not only mood but also stress level [84]. Subjects who listen to soft music commonly report an improvement in mood and a pleasure sensation with increased levels of serotonin and decreased cortisol levels [85]; conversely, high intensity and short sounds are associated with an increase in noradrenaline that stimulates hyper mood activity [86].

Although rare, diplacusis was observed in both PR and CL PMs, although it was more common among CL PMs. Diplacusis is a perceptual anomaly whereby the same sound is perceived as having a different pitch depending on whether it is presented in the left or the right ear; this phenomenon is common in individuals with asymmetric hearing loss [25]. The symptom can be due to a shift of the excitation peak from a high to a more basal region of the basilar membrane following a cochlear damage [87] which in turn causes a shift in the peak of the neural tuning curves of the auditory neurons toward lower frequencies [88] or to the fall of a specific frequency inside a dead cochlear region that evokes an unclear stimulus [89]. The limited number of studies that investigated this symptom included in this review does not allow a statistical analysis and comparison to other symptoms, but only the description of the phenomenon.

### 4.4. Limits of Our Study

Our study has some limits. First, the studies included in the review had a high degree of heterogeneity, especially for methodologies, evaluation of HL prior to exposure to music and outcome reporting. Secondly, we considered HL as any pure tone threshold higher than 25 dB; however, definitions of HL in the included studies were not consistent. Furthermore, many studies did not report the entity of HL; therefore, in our study we could not describe the entity of HL and could only consider it as present/absent and symmetric/asymmetric. Associated symptoms have not been investigated in all the studies included in the review; therefore, the prevalence of the symptom could only be calculated for the studies that investigated it. Heterogeneity between studies also affected, even if at a lesser extent, the definitions of tinnitus, diplacusis, and hyperacusis. Taken together, these limits prevent the execution of a meta-analysis and should be considered when interpreting the results and the conclusions of our review.

### 4.5. Future Perspectives for Early Diagnosis of Hearing Damage in Professional Musicians

The use of individual hearing protection devices and strict regulations for time and entity of noise exposure is of paramount importance for the prevention of HL and associated symptoms in PMs. Future perspectives in noise-induced HL risk prevention are centered on early diagnosis of hearing impairment in predisposed individuals. On this topic, some authors recently proposed the use of microRNAs for monitoring the hearing damage [90] because they allow to investigate the hearing pathways from periphery (34a, 29b, 76, 96 and 431) to central auditory area (miR − 9/9 *). MicroRNA are very sensible to the cells metabolic alterations and they are commonly used for monitoring the progression of several disease [91,92]; furthermore, they have shown a high sensitivity in the early identification of inner ear cellular damage [93] and could play a role in the early diagnosis of hearing damage in individuals that are at risk of HL.

## 5. Conclusions

Our systematic review showed that HL is common in PMs, and PR PMs are at higher risk of developing HL compared to CL PMs. These findings could be explained by the prolonged exposure to high-frequency and high-intensity sounds common in PMs, that more often occurs in PR PMs. The prevalence of asymmetric HL was significantly higher in CL PMs compared to PR PMs, probably due to the type of instruments used. Tinnitus was equally present in PR and CL PMs; hyperacusis was more prevalent in PR PMs and diplacusis was more common in CL PMs. The use of individual hearing protection devices may help reduce the risk of noise-induced HL and associated symptoms in PMs; furthermore, the monitoring of microRNAs concentration associated with traditional hearing tests might play a role in the early diagnosis of hearing damage in exposed individuals that are at risk of HL.

## Figures and Tables

**Table 1:** Articles included in systematic review.

Author, Year	Design	Subjects (*n*)	Age (Range)	Music Genre	Rehearsal Time (hs)	HL (%)	HL (Frequency)	HL (Symmetry)	Tinnitus (%)	Hyperacusis (%)	Diplacusis (%)
Axelsson, 1978 [33]	PS	83	ns	PR	1:00	30%	3000–6000 Hz	Symmetric	ns	ns	ns
Axelsson, 1981 [34]	PS	38	27–33	PR	4:30	28.9%	4000–6000 Hz	Symmetric	ns	ns	ns
Axelsson, 1981 [35]	PS	139	20–69	CL	ns	43.2%	4000–8000 Hz	Symmetric	ns	ns	ns
Karlsson, 1983 [36]	LS	392	20–69	CL	ns	25%	6000–8000 Hz	Asymmetric	ns	ns	ns
Johnson, 1986 [37]	CC	60	24–64	CL	ns	none	ns	ns	ns	ns	ns
Royster, 1991 [38]	PS	59	30–69	CL	2:30	52.5%	6000 Hz	Asymmetric	ns	ns	ns
Axelsson, 1995 [39]	PS	83	20–46	PR	3:30	10.8%	3000–6000 Hz	Symmetric	12%	9%	ns
Obeling, 1999 [40]	PS	57	22–65	CL	ns	17.5%	4000–8000 Hz	Asymmetric	ns	ns	ns
Kahari, 2001 [41]	PS	140	23–64	CL	ns	52.5%	6000–8000 Hz	Asymmetric	ns	ns	ns
Kahari, 2001 [42]	LS	56	30–50	CL	ns	76.8%	4000–8000 Hz	Symmetric	ns	ns	ns
Eaton, 2002 [30]	PS	53	25–60	CL	3:00	24.5%	3000-6000 Hz	Symmetric	ns	ns	ns
Kahari, 2003 [43]	CSS	139	26–51	PR	5:00	74.1%	3000–8000 Hz	Symmetric	48%	45%	2.8%
Mendes, 2007 [29]	PS	34	ns	PR	ns	58.8%	3000–6000 Hz	Symmetric	47%	ns	ns
Beltrao Amorim, 2008 [44]	PS	30	18–40	PR and CL	3:50	16.7%	3000–6000 Hz	Symmetric	ns	ns	ns
Sayegh, 2008 [45]	PS	340	18–28	CL	ns	63.8%	ns	Asymmetric	28%	ns	ns
Jansen, 2009 [46]	PS	241	23–64	CL	ns	51.9%	6000 Hz	Symmetric	17%	ns	8.2%
Hasson, 2009 [47]	CS	250	ns	CL	ns	6%	ns	ns	19%	14%	ns
Phillis, 2010 [48]	PS	329	18–25	PR and CL	2:00	45%	4000–6000 Hz	Symmetric	ns	ns	ns
Pawlaczyk, 2011 [49]	PS	127	22–67	CL	4:30	26%	2000–4000 Hz	Symmetric	ns	ns	ns
Toppila, 2011 [50]	PS	63	22–52	CL	ns	100%	4000–6000 Hz	Symmetric	9.5%	6.3%	ns
Samelli, 2012 [51]	CC	16	21–41	PR	3:15	100%	2000–3000 Hz	Symmetric	ns	ns	ns
Raymond III, 2012 [52]	PS	32	35–64	CL	ns	25%	4000–8000 Hz	Symmetric	ns	ns	ns
Patil, 2013 [53]	CC	84	26–47	CL	ns	none	ns	Symmetric	ns	ns	ns
Russo, 2013 [54]	PS	44	41–57	CL	4:00	100%	4000–6000 Hz	Symmetric	ns	ns	ns
Goncalves, 2013 [55]	CS	50	21–51	CL	4:00	32%	2000–16,000 Hz	ns	ns	ns	ns
Wilson, 2013 [56]	PS	144	18–60	CL	3:15	22.9%	4000–8000 Hz	Symmetric	ns	ns	ns
Luders, 2014 [57]	RS	42	18–58	CL	ns	7.14%	250–3000 Hz	Symmetric	ns	ns	ns
O’Brien, 2014 [58]	PS	367	35–51	CL	ns	42.5%	2000–8000 Hz	ns	34%	ns	ns
Schmidt, 2014 [59]	CC	212	20–69	CL	4:00	60.8%	3000–6000 Hz	Asymmetric	ns	ns	ns
Halevi-Katz, 2015 [31]	PS	44	20–64	PR	5:15	100%	3000–6000 Hz	Symmetric	6%	2%	ns
Dudarerewicz, 2015 [60]	PS	18	30–58	CL	3:30	27.8%	4000 Hz	ns	ns	ns	ns
Stormer, 2015 [61]	CC	111	16–52	PR	2:00	37.8%	6000 Hz	Symmetric	10%	ns	ns
Luders, 2016 [62]	PS	30	33–54	CL	3:00	43.3%	3000–6000 Hz	Symmetric	53%	33%	ns
Hennir, 2016 [63]	PS	28	18–25	CL	2:00	25%	ns	Symmetric	ns	ns	ns
Luders, 2016 [64]	PS	100	28–38	PR and CL	ns	32%	ns	ns	ns	ns	ns
Pouryaghoub, 2017 [65]	CS	125	31–38	PR and CL	ns	82.4%	3000–6000 Hz	Asymmetric	51.2%	ns	ns
Hoydal, 2017 [28]	CC	111	16–52	PR	ns	100%	1500–6000 Hz	Asymmetric	19.8%	ns	ns
Pawlaczyk-Luszczynska, 2017 [66]	CC	168	18–29	CL	4:00	13.1%	6000 Hz	Symmetric	32.1%	27.4%	ns
Stormer, 2017 [67]	CS	111	22–41	PR	ns	ns	ns	ns	19.8%	ns	ns
Szibor, 2018 [68]	PS	22	18–62	PR	ns	95.4%	6000 Hz	ns	27.3%	27.3%	ns
Behar, 2018 [69]	LS	46	ns	CL	ns	100%	4000–8000 Hz	ns	ns	ns	ns

List of articles included in our systematic review. For each article, we specified: first author and year, type of study, number of subjects included, age range, type of music played by study participants, rehearsal time, percentage of hearing loss, affected frequencies, symmetry of hearing loss, percentage of audiological symptoms (tinnitus, hyperacusis, diplacusis). PS: prospective study; LS: longitudinal study; CS: cohort study; CC: case-control study; CSS: cross-sectional study; RS: retrospective study. PR: pop/rock; CL; classical. ns: not specified.
